# Evaluation of Human Anti IgG Polyclonal Antibody Production Conjugated with Peroxidase in Egg Yolk

**Published:** 2019-07

**Authors:** Fahimeh ABDOLMALEKI, Zahra ZAMANI, Somayeh TALEBI

**Affiliations:** 1. Department of Biochemistry, Tehran East Branch, Payam Noor University, Tehran, Iran; 2. Department of Biochemistry, Pasteur Institute of Iran, Tehran, Iran; 3. Department of Microbiology, Faculty of Advanced Science and Technology, Tehran Medical Sciences, Islamic Azad University, Tehran, Iran

**Keywords:** Egg yolk, HRP, Human serum, IgY, Polyclonal antibody

## Abstract

**Background:**

Egg yolk is a rich and accessible source of yolk immunoglobulin (Y immunoglobulin). Presently, polyclonal antibodies from mammalian sources are used for diagnosis. Antibody production from egg yolk gives a higher yield and turnover than that from lab animals, and invasive methods such as phlebotomy and causing stress to the animals are not required. Due to the issues regarding mammalian antibodies, we aimed to evaluate the human anti-IgG polyclonal antibody production conjugated with peroxidase in egg yolk.

**Methods:**

Population of laying hens reared in Agriculture/Isfahan University of Technology were used in 2017. After immunizing hen against pure human IgG, specific IgY (yolk immunoglobulin) was purified from the yolk by sedimentation with polyethylene glycol (PEG6000). To assess the molecular weight and activity of the product, SDS-PAGE and ELISA-test were used, respectively.

**Results:**

The complete molecular weight of IgY was 180 kDa and the molecular weight of its light and heavy chains were 27 and 67 kDa, respectively.

**Conclusion:**

Antihuman IgG IgY had a purity above 90%. The product of this study can be used to measure IgG class antibodies in order to diagnose different diseases.

## Introduction

IgG is the most abundant immunoglobulin present in the plasma and constitutes 80% of all immunoglobulins (1). IgG is the most important isotype of immunoglobulin in the secondary immune response. Hens IgG is called IgY (yolk immunoglobulin), which can enter the egg and protect the fetus until it hatches (2,3). Among the medical applications of IgG is the second test for evaluation of patients with symptoms of humoral immune system deficiency or compound immune deficiency (cellular or hormonal) (4). In patients with symptoms of possible immune system deficiency with either hypogammaglobulinemia or a normal concentration of the total IgG, measurement of the subclasses of IgG may be useful (5). Since several years multiple studies have been carried out for using IgGs in medical diagnosis (6,7), and treatment for working against certain types of infectious agents, in particular, intestinal pathogens (8–10). Considering the increasing importance of IgG, researchers have found various methods to produce this type of antibody in hens and to purify them from egg yolk (3, 11, 12). IgG immunoglobulins of one species are immunogenic for other species (13). By injecting the human antibodies in other animals, their antiantibodies can be obtained and used for research and treatment (14). Our purpose was to produce the antihuman IgG conjugated with peroxidase in egg yolk. The IgY in the eggs acts specifically against the antigen (IgG) injected in the hen and is produced to immunize the hens against the antigen (15,16).

The antibody is produced and collected easily on a daily basis without any invasive methods such as phlebotomy. This antibody can be stored in the egg yolk at 4 °C for at least a year. IgY is rather resistant to heat and acid (17–19).

Our objective was to find an antibody to replace the mammalian antibody, and by choosing an appropriate and economic method, to isolate and purify IgY from egg yolk.

## Materials and Methods

### The hen’s immunization

Population of laying hens reared in Agriculture/Isfahan University of Technology were used in 2017. These hens were randomly divided into two groups of 20 hens each. First group considered as a control group in which no treatment was given, second group considered as an experiment group, immunized. The egg-laying hens of the experimental group were injected with the immune solution, in two different muscles of the chest. The IgG antigen, with few minor changes, was emulsified along with complete Freund adjuvant (Merck, Germany) (20). Samples (consisting of eggs and hens blood) were collected from the experimental group egg-laying hens from week 0 (before immunization) until week 10 after immunization (21).

### Isolation of proteins from the lipids of egg yolk

The eggs were broken and the yolk, egg white, and the membrane around the yolk were separated (22,23). The volume of egg yolk, PBS (phosphate-buffered saline) 1× buffer, and 12% polyethylene glycol 6000 (Merck, Germany), was added to the solution twice. The tubes were placed on a rolling apparatus for 10 min; thereafter, the solutions in the tubes were homogenized by vortexing, and then, the solution was centrifuged for 20 min at 10,000 rpm at 4 °C. The supernatant was passed through a filter by using Buchner and a suction pump to obtain a clear solution. Polyethylene glycol 6000 was added to the clear solution, followed by rolling after 10 min. The solution was vortexed and was centrifuged twice at 4 °C for 20 min at 11,952 g. Herein, the supernatant was dispensed and the pellet was resuspended with 10 ml PBS 1× and 5% polyethylene glycol 6000 was added. The solution was rolled for 10 min, followed by vortexing. Centrifugation was carried out for 20 min at 11952 g in 4 °C. The supernatant was dispensed and the pellet was resuspended with 1 ml PBS 1×. A dialysis sack was boiled and the solution along with PBS 0.1× buffer was poured into the sack and was stirred on a stirrer in a cold room for one night. The next morning, the salt buffer was dispensed and the sack was added into PBS 1× and was further stirred for 3 h. The solution in the sack was poured into a microtube and was stored at −20 °C.

### Determining the protein concentration in the aqueous solution

The concentration of the protein present in the aqueous solution was measured via a spectrophotometer apparatus. Initially, the apparatus was calibrated with PBS 1× at a wavelength of 280 nm. One hundred μl of the pure protein solution was mixed with 900 μl of PBS 1× and its absorption was read at 280 and 260 nm, respectively.

Concentration measurement formula:

Protein concentration (mg/ml)=(absorbance at 260 nm×0.76)−(absorbance at 280 nm×1.55)

### Immunodiffusion assay

The specificity of IgY against IgG was demonstrated with the agar diffusion test (1% agarose) (24, 25). For this test, percent agar gel was prepared as following: 1.2 g agar was dissolved in 100 ml of 0.01 M PBS and the solution containing agar was boiled in an electric heater until the agar was well dissolved in the solution; thereafter, the obtained solution was poured into a petri dish and six wells (one in the center and five in the outer corners) were created into the gel. Antigen was poured into the center well, whereas in the outer corner wells, crude solution and dilutions of 1:2, 1:4, 1:8, and 1:16 of antibody and PBS buffer were poured; incubation was done for 24 h, following which the incubation the gel was stained with Coomassie blue for 30 min, and then with a decolorizing solution. Eventually, clot formation between the central well and different dilutions of the antibody were examined carefully.

### Polyacrylamide gel electrophoresis in the presence of sodium dodecyl sulfate

SDS-PAGE was carried out according to the coverslip method using a 10% isolating gel and a 4% condensing gel (26). Fifty μl volume sampling buffer was added to 50 μl volume sampling protein. In one instance, buffer without 2ME was used, whereas, in next instance, time buffer with 2ME was used. Ten, 20, and 40 μl buffer and sample were added to the wells. One hundred μl of the sampling buffer along with 100 μl of the purified antibody was boiled and 10, 20, and 40 μl of the same were added to columns 2, 3, and 4, respectively, using a Hamilton syringe; the first column was dedicated to the protein marker. Electrophoresis was done in a steady electrical current with the voltage of 80 mA. After electrophoresis, the gel was stained with Coomassie blue.

### Measurement of IgY activity via ELISA-test

The activity of the anti-IgG antibody was measured with the ELISA method (27, 28). The reaction ended 20 min after adding 100 μl of 5% sulfuric acid and the absorption of the wells at 450 nm was read by ELISA reader.

The first two rows of the wells were negative controls and lacked the first antigen for coating; in the other wells, 2 μg/ml antigen was coated.

### Conjugation of IgY with HRP enzyme

Conjugation of IgY with HRP was performed according to Wilson and Nakane (29).

## Results

### Specific IgY in sera

**Fig. 1:**
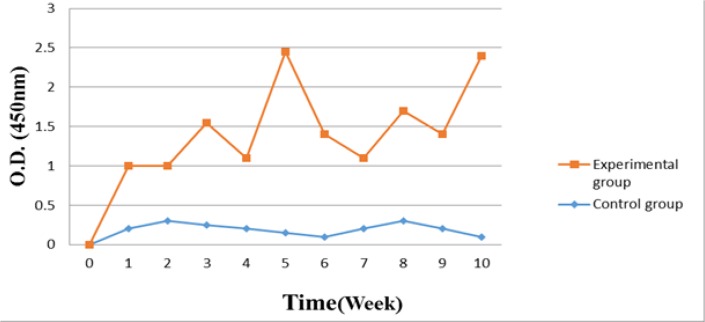
The changes of specific IgY titer in serum obtained from laying hens immunized with anti-IgG IgY and control group during the immunization period

Specific anti-IgG IgY were detected in the sera of immunized hens (experiment group) in first week after the initial immunization. The booster immunizations levels of specific IgY were increased significantly and remained relatively high throughout the experiment period, as shown in Fig. 1.

### Immunological identification of IgY’s crude extract

The Ouchterlony of the antibodies is illustrated in Fig. 1; IgG was added to the central well, and the serial dilutions of the crude extract were added to the peripheral wells, respectively.

**Fig. 2:**
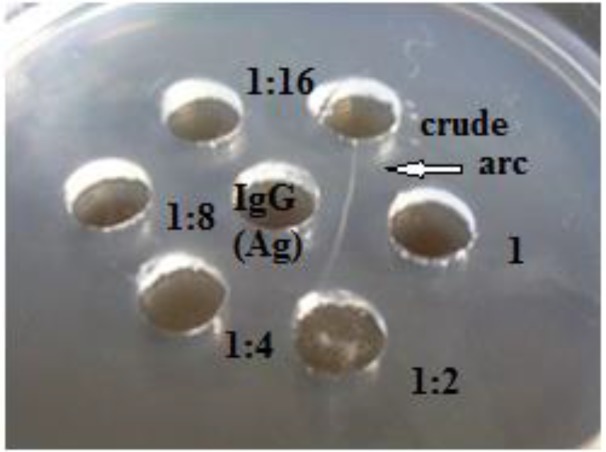
Immunological assessment of anti-IgG IgY In the central well: IgG. In the peripheral wells: anti-IgG IgY crude extract (1200 μg); the serial dilutions (1:2, 1:4, 1:8, and 1:16, respectively). Halo formation (arc) in the parts that antibody and antigen have met in agarose gel

The white precipitation lines occurred between the central well, whereas each peripheral well indicated the immunological activities of IgY (Fig. 2). The titer of the crude extract by immunodiffusion was about 1:16.

### Extraction, purification, and biochemical identification

**Fig. 3:**
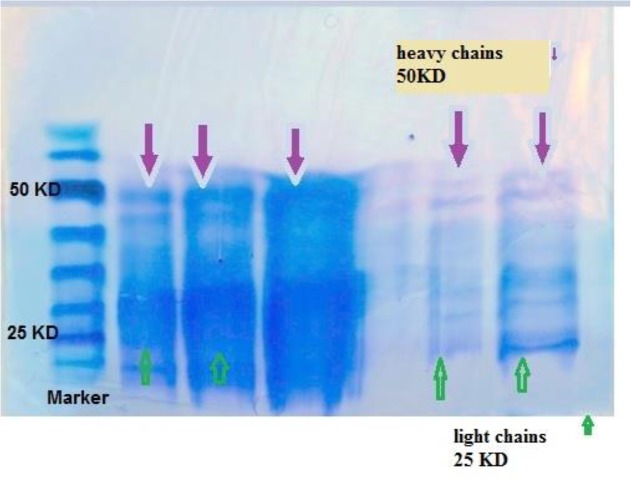
Analysis by SDS-PAGE in reduced conditions of IgY, after isolation and purification process. SDS-PAGE on 10% gel. Lane 1: protein marker on SDS-PAGE. Electrophoresis gel, lane 2, 3, 4, 5, 6: purified IgY from egg yolk and band formation in 50 and 25 kDa parts, which each is due to the heavy and light chains of IgY, respectively

Production of anti-IgG antibodies, isolated from the lipids of the yolk via sedimentation with PEG 6000, solubilized the proteins of the yolk; with centrifugation, the proteins of the yolk were purified. The purity of IgY reached approximately 90%. SDS-PAGE revealed that the method used for IgY purification was suitable. Figure 3 illustrates the antibody purified from the egg (purification by organic solution) with sampling buffer with 2 ME; 2 ME separates the heavy and light chains of IgY from each other and the bands formed in 50 kDa and 25 kDa are due to the heavy and light chains, respectively.

**Fig. 4:**
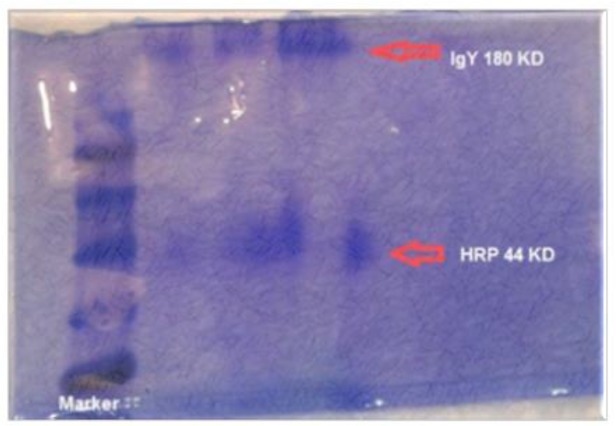
SDS-PAGE (nonreducing) on 10% gel of anti-IgG IgY-HRP conjugate. Lanes (1): proteinladder (2): anti-IgG IgY-HRP conjugate. Gel electrophoresis band formation in the 44 and 180 kDa parts, which represent IgY and HRP, respectively

The final concentration of anti-IgG IgY-HRP conjugate was 1 mg/ml. The conjugation between IgY and HRP was confirmed by SDS-PAGE. The result revealed that two bands of 180 KDa and 44 KDa appeared, with no other contamination bands (Fig. 3). These two bands belonged to IgY and HRP, respectively. Figure 4 illustrates the band of the antibody purified from hen serum and conjugated with peroxidase enzyme with sampling buffer without 2 ME. The molecular weight of the bands is approximately 180 kDa, which proves the presence of IgY.

### ELISA test

**Fig. 5:**
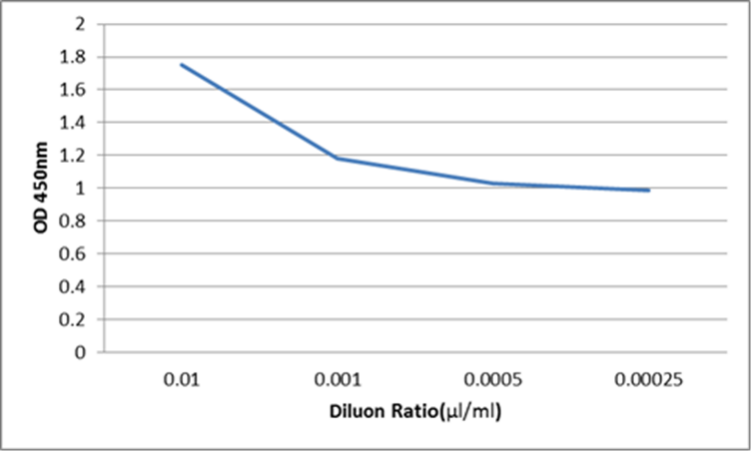
The activity of serial dilution of IgY antibody isolated from egg yolk conjugated with HRP against human IgG

**Fig. 6:**
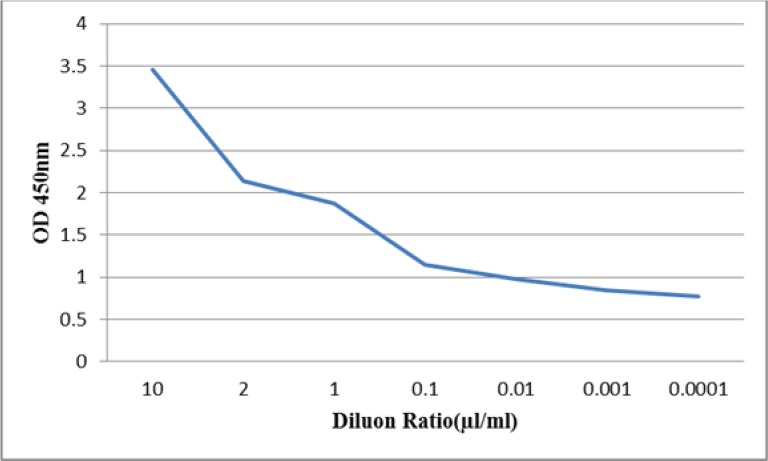
The activity of serial dilution of IgY antibodies isolated from egg yolk against human IgG

In the ELISA sandwich test, the yellow color produced at the end of the experiment indicates the formation of antibody complex and a positive result. The antibody conjugated with peroxidase acts against IgG until 1:4000 dilution (Fig. 5). The results of direct ELISA indicate that the antibody acts against IgG until 1:10,000 dilution (Fig. 6).

## Discussion

For IgY purity, antihuman IgG was more than 90%. Moreover, similar to IgG, IgY is composed of two heavy chains and two light chains; however, with a molecular mass of 180 kDa, the molecular weight of IgY is larger than that of mammalian IgG (150 kDa). The H-chain of IgY has a larger molecular mass than that of IgG (30). Production of anti-IgG IgY is essential to detect and assess the activity of IgY and to design immunochemistry kits. A refined IgY extraction method could improve the quality of IgY product and make a breakthrough in IgY technology (31). In this study, injection of IgG along with Freund adjuvants to hens induced the production of anti-IgG antibodies, isolated from the lipids of the yolk via sedimentation with PEG 6000 that solubilized the proteins of the yolk; with centrifugation, the yolk proteins were purified. The Ouchterlony method in this study was carried out to identify and assess the activity of the antibodies. The results indicated clotting between IgG and IgY; the purity and activity of IgY against IgG were also confirmed by SDS-PAGE and ELISA, respectively.

For the wide utilization of IgY, various methods, such as WD method, caprylic acid method, chloroform (CF) method, phenol (PHE) method, carrageenan (CARRA) method (32–34), have been developed to improve the purity and quantity of the IgY. For example, the purity of IgY could reach 98.3% and yield of 73% by sodium sulfate precipitation (22). Affinity chromatography was used with a synthetic ligand (TG19318) to purify IgY and they obtained a product with 90% purity (35). A refined chromatography involving human mycoplasma protein (protein M) was reported to recover extremely high purity (98.7%) of polyclonal IgY from the water-soluble fraction (WSF) by retaining the functional activity (36). Dilution with acidic distilled water is a simple and applicable way to isolate the hydrophilic proteins including IgY from egg yolk (37). In another study, affinity chromatography was used; after salting out with ammonium sulfate, the yield was reported to be 81% (37). A certain amount of dextran sulfate and calcium chloride were added to WSF centrifuged by 4-fold Tris-HCl buffer. After centrifugation, the precipitate was removed and the supernatant was mixed with sodium sulfate. The purity of IgY reached approximately 80% (38). In this study, sedimentation with PEG was done according to Palson’s protocol and the part containing IgY sedimented appropriately (39). One of the benefits of using PEG for sedimentation of proteins is the ability to use it in temperatures above 0 without denaturing the protein. Furthermore, in this method, unlike the salting out method, there is no need for dialysis afterward and the rest of the purification can be carried out with centrifugation; however, notably, the yield of IgY isolation only by the Palson method is low and a large portion of IgY is wasted, although, after centrifugation, which is the final part of isolation, IgY has a high purity and yield (40). The purity of IgY extracted by our method could reach 90%. PEG precipitated procedure could improve the antibody quality in purity and concentration, which meets the requirements for producing high-quality IgY. Moreover, this study reported that the complete molecular weight of IgY is about 180 kDa and its light and heavy chains weigh about 25 and 50 kDa, respectively. Our approach simplified the entire isolation procedure, leading to less time consumption and improved purity.

IgY is the major low molecular weight immunoglobulin in oviparous animals (41). This type of antibody has distinctive properties, explored in various ways in research, diagnostics, and therapy. One important advantage arises from the phylogenetic distance and genetic background that distinguishes the birds from mammals (42) Since polyclonal IgY can be recovered from the eggs of laying hens for prolonged periods, this approach provides a long-term supply of substantial amounts of antibodies (43,44). Furthermore, the aspect of animal welfare is important since the antibodies are noninvasively extracted from egg yolk (45). These results and the benefits of IgY enable IgY to be used as an abundant and suitable source of polyclonal antibodies, used for several purposes providing the final IgY preparations fully acceptable for various human applications, such as food additives, peroral medications or cosmetics, and multiple uses in medical diagnosis.

The relatively appropriate yield of this study reveals that with the appropriate immunization of hens, the proportion of specific IgY to total IgY can be increased. The procedure of isolation was simplified with a higher yield of IgY, avoiding energy- and time-consuming methods such as ultrafiltration or use of chromatography.

## Conclusion

We used PEG method, to isolate IgY from yolk. Considering several factors (cost, ease of use, purity, yield, safety etc.), the production of antibodies in hens and the IgY-extraction by means of PEG-precipitation is very cost-effective and results in highly specific antibody with stable titers up to 1:1,000,000. Due to the phylogenetic distance between Aves and Mammalia, chicken is able to produce specific antibodies against highly conserved mammalian antigens. The extraordinary amount of antibody obtained by IgY-technology also encourages using IgY-Ab in human- and veterinary medicine for therapeutic/prophylactic purposes.

Immunoglobulin IgY from egg yolk is taken to produce a mass IgG protein detection kit. Other immunoglobulins IgY are used in medical, pharmaceutical and nutritional research. Investigations should be done in cancer patients for diagnosis and treatment.

## Ethical considerations

Ethical issues (Including plagiarism, informed consent, misconduct, data fabrication and/or falsification, double publication and/or submission, redundancy, etc.) have been completely observed by the authors.
